# ID 225 - Monitoramento do Horizonte Tecnológico (MHT) de Inibidores de Janus Quinase (JAKi) para o Tratamento de Lúpus Eritematoso Sistêmico (LES)

**DOI:** 10.5327/2237-9622.2025.v34s1.225

**Published:** 2025-11-25

**Authors:** Luiza Leal do Nascimento Costa, Natália Brandão, Renato Mantelli Picoli, Renata Ferreira, Júlia Lima, Pedro Antonio Barbosa, Aline Rocha, Hellen Miyamoto

**Keywords:** JAKi, lúpus eritematoso sistêmico, monitoramento do horizonte tecnológico

## Abstract

**Introdução:**

O LES é caracterizado como uma doença autoimune com manifestações complexas e multissistêmicas, com opções terapêuticas limitadas. O bloqueio das vias de sinalização JAK está sendo investigado no controle da inflamação subjacente ao LES, trazendo novas perspectivas para o tratamento. Atualmente, nenhum JAKi tem registro para tratamento de LES na Agência Nacional de Vigilância Sanitária (Anvisa), na Food and Drug Administration (FDA) e na Agência Europeia de Medicamentos (EMA). Este MHT visa identificar sistematicamente os JAKi em desenvolvimento para o tratamento do LES.

**Método:**

Foram recuperados registros de ensaios clínicos nas bases ClinicalTrials.gov, WHO International Clinical Trials Registry e Cochrane Central Register of Controlled Trials. Foram utilizadas estratégias de busca com vocabulário controlado e sinônimos relacionados aos JAKi e ao LES. Não foram aplicados filtros de restrição temporal, idioma ou status do estudo. Ensaios clínicos de fase 2 e 3 foram incluídos, enquanto estudos observacionais foram excluídos.

**Resultados:**

Foram identificados sete JAKi em desenvolvimento para o tratamento de LES. Baricitinibe, indicado em LES ativo, é um inibidor de JAK1 e JAK2, avaliado nos ensaios NCT03616912 e NCT03616964 (fase 3, completos e com resultados). Filgotinibe, indicado para nefropatia membranosa lúpica e LES cutâneo, é um inibidor seletivo de JAK1 de segunda geração, testado nos estudos NCT03285711 e NCT03134222 (fase 2, completos e com resultados). Upadacitinibe, indicado para LES ativo moderado a grave, é outro inibidor seletivo de JAK1, avaliado nos ensaios NCT03978520 (fase 2, completo e com resultados) e NCT04451772 (fase 3, sem recrutamento). Deucravacitinibe, inibidor alostérico de TYK2, é indicado para LES ativo moderado a grave e nefrite lúpica, com ensaios NCT03252587 (fase 2, completo, com resultados), NCT03920267 (fase 2, sem recrutamento), e os ensaios NCT05617677 e NCT05620407 (fase 3, em recrutamento). Brepocitinibe, um inibidor seletivo de TYK2 e JAK1, foi testado em pacientes com LES ativo moderado a grave que falharam ao tratamento padrão, no estudo NCT03845517 (fase 2, completo e sem resultados). Tofacitinibe, destinado a pacientes com envolvimento cutâneo moderado a grave, é um inibidor de JAK1, JAK2 e JAK3, com menor efeito sobre TYK2, avaliado no estudo NCT03288324 (fase 2, completo, sem resultados). Por fim, solcitinibe, um inibidor seletivo de JAK1, foi testado em LES ativo no estudo NCT01777256 (fase 2), mas o ensaio foi encerrado precocemente após a primeira análise interina, devido à ausência de redução no desfecho primário.

**Conclusão:**

Os estudos com baricitinibe não conseguiram atingir desfechos importantes, como a redução gradual da dose de glicocorticoides e o tempo até o primeiro surto grave, levando à interrupção do estudo. Da mesma forma, o desenvolvimento do solcitinibe foi descontinuado após as análises interinas indicarem que o desfecho primário não foi alcançado. Por outro lado, outros JAKi (como filgotinibe, upadacitinibe, deucravacitinibe, brepocitinibe e tofacitinibe) continuam sendo avaliados em ensaios clínicos. O desenvolvimento dos JAKi traz novas possibilidades para o tratamento da LES, sendo uma estratégia promissora. No entanto, ainda se faz necessário investigar quais grupos com LES realmente se beneficiariam com os iJAK.

